# Antepartum hemorrhage due to placenta previa with autologous transfusion: A case report

**DOI:** 10.1016/j.ijscr.2024.109999

**Published:** 2024-07-04

**Authors:** Rizki Dunniroh Kaukaba, Septian Adi Permana, Arif Zuhal Amin Hananto, Faizal Muhammad

**Affiliations:** Anesthesiology and Intensive Care Department, RSUD Dr. Moewardi, Faculty of Medicine, Universitas Sebelas Maret, Surakarta 57126, Indonesia

**Keywords:** Antepartum hemorrhage, Blood transfusion, C-section, Delivery, Placenta previa

## Abstract

**Introduction and importance:**

Bleeding during pregnancy or during childbirth can cause significant morbidity and mortality for the mother and baby, this can be overcome by performing a caesarean section (C-section) and blood transfusions. Although blood transfusions can save lives, there is a risk such as transfusion reactions, transmission of infection, and anaphylaxis. Giving autologous blood transfusion can reduce the risk of these events. This case report aims to investigate the advantages of autologous blood transfusion in managing the patient's hemodynamic status compared to homologous blood transfusion.

**Case presentation:**

A 21-year-old female (G2P1A0) with antepartum hemorrhage (APH) due to placenta previa underwent emergency C-section with intrauterine device installation and hysterectomy. The patient received an autologous transfusion to improving the hematocrits and hemoglobin within 30 min. Autologous transfusion provided routine postoperative hemodynamics, electrolytes, and blood stability. However, it could not completely replace homologous transfusion.

**Clinical discussion:**

Autologous transfusion reduces transfusion response, infection risk, and immunosuppression. Consequently, it reduces the need for allogenic blood supplies and enables safer transfusion for people with rare blood types and various auto-antibodies.

**Conclusion:**

Autologous transfusions may provide better outcomes in C-section surgery for APH patients due to placenta previa. Thus, we recommend the use of autologous over homologous transfusion. Further research is required to compare them to a large population.

## Highlights

• Reinfusion of blood or blood components into the same person from whom they were taken is known as autologous blood transfusion.
• Placenta previa occurs with an incidence of 0.3-0.5% of all pregnancies. Placenta previa is also found in 20% of APH which can cause death in mothers and newborn.
• Autologous transfusion provided better postoperative C-section and increased RBC, Platelets, HCT, and Hb compared to homologous transfusion

## 1 Introduction

Antepartum hemorrhage (APH) is characterized by vaginal hemorrhage during the second half of pregnancy. Its incidence is a cause of perinatal death and maternal morbidity worldwide [1]. Placenta previa and placental abruption account for 0.5–5 % of APH [2]. APH is associated with adverse maternal complications ranging from preterm delivery, postpartum hemorrhage, shock, sepsis, retained placenta, increased caesarean section (C-section) rates, peripartum hysterectomy, coagulation failure and even death [3]. Furthermore, it can cause low birth weight, intrauterine fetal death and birth asphyxia [4].

Patients with placenta previa are at increased risk for blood loss during C-section, therefore preparation for blood transfusion is required. Although blood transfusions can save lives, there are significant risks associated with homologous blood transfusions. It has been demonstrated that homologous transfusions are a separate risk factor for post-operative infection and may be the primary risk factor for postoperative bacteremia. Thus, autologous transfusion, also known as cell saver, can become as alternative approach [5].

In a homologous transfusion, someone's compatible donor's blood is obtained and transfused into other recipient within same species. Meanwhile, the process of gathering and re-infusing a patient's own blood or blood components is known as autologous blood transfusion [6]. The use of autologous may increase erythrocytes, platelets, hematocrit, and hemoglobin, however decrease IgA, IgG, and IgM. Thus, autologous blood transfusion can be an option in C-section with placenta previa [6]. Several studies have shown storage of autologous blood during pregnancy to be relatively safe for the mother and childbirth and to be a feasible and reasonable transfusion practice [3].

The management of APH requires firm cooperation from all health sectors, blood transfusion preparation, operator preparation, and skilled anesthesiologists to reduce unfavorable maternal and perinatal outcomes [7]. Because more autologous blood must be extracted than the median amount usually required to prevent further allogeneic transfusions, up to half of the blood that is collected may be wasted. Due to the high health standards, most autologous donors do not meet the criteria for using leftover blood for other patients. The collection costs are higher than for autologous transfusion due to this blood waste and the expenses associated with running autologous programs. Other dangers include ABO hemolytic reactions to the transfusion brought on by administrative or clerical errors, volume excess, and bacterial infection [8,9]. Despite those cost-handling difficulty and lack of autologous transfusion availability, we considered to use autologous transfusion as the live-saving and alternate approach over homologous blood transfusion due to autologous transfusion provide better prognostic survival rate [10]. Thus, it is important to set a case report to describe the advantage and disadvantages of autologous transfusion. This case has been written according to latest Surgical CAse REport (SCARE) Guidelines [11].

## 2 Presentation of case

A 21-year-old female, weighing 81 kg, with a G2P1A0 pregnancy was diagnosed as APH due to placenta previa. She bled approximately 1 sanitary pad (±50 cc). Her gestational age was 36 weeks with one C-section history. The fetal heart rate was 139 bpm with active fetal movement. She was admitted to the operating room for an emergency C-section, intrauterine device installation, and hysterectomy.

Physical examination showed a clear airway, 20 times per minute respiratory, and normal pulmonary auscultation. Hemodynamics status showed 100 % pulse oximeter with 3 l per minute nasal cannula, normal capillary refill, and no sign of congestion. Vital signs showed blood pressure 140/90 mmHg, heart rate 87 bpm, and 36.1 °C of axillary temperature. The patient was conscious without any focal neurological deficit.

Laboratory tests showed Hb 9.9 g/dL, Hct 32 %, WBC 10,2/μL, Albumin 2.8, and HS-CRP 9.52 mg/l. The anesthesia evaluation found several issues such as BMI 32.8 kg/m^2^, STOPBANG score 0, and insufficient meal fasting and blood supply of 4 PRC, 4 whole blood, 4 thrombocyte concentrate, and 4 FFP.

Before entering the operating room, the patient received 75 mg of 5 % Lidocaine HCl injection. She also received general anesthesia injections such as Midazolam 2 mg, Fentanyl 50 μg, Propofol 100 mg, and Atracurium 50 mg. Then, she was intubated with endotracheal tube No. 7.0 (level 20 cm) and the help of a ventilator. We decided to use autologous transfusion because her preoperatively Hb was low in her emergency case and we anticipated >1 l of blood loss. The perioperative blood loss was 1500 cc. She received a total of 1800 cc autologous transfusion, 1000 cc Crystalloid, and 500 cc Colloid infusion. The C-section was performed for 3.5 h with placenta accreta finding. The 3260-gram male baby was born with an Apgar score of 4/5/6.

**Fig. 1:**
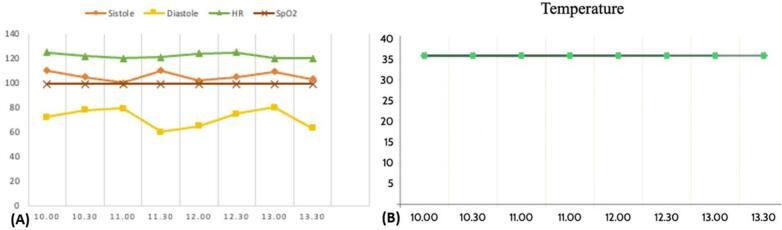
Vital signs profile of the patient during the c-section procedure. (A) Graph of hemodynamics during surgery, (B) Graph of temperature during surgery.

The patient's hemodynamics are monitored continuously during C-section. Her blood pressure and temperature were stable without significant heart rate changes (Fig. 1-A and Fig. B). Post-surgical laboratory profile showed Hb 11.3 g/dL, Hct 37 %, WBC 12,240/μL, platelets 201,000/μL, PPT 12.9 (N: 9.3–11.4), aPTT 30.0 (N: l 24.5–32.8), and INR 0.95.

The patient was observed in the ICU with mechanical ventilator support. Hemodynamic status showed blood pressure 110/68, MAP 82 mmHg, heart rate 115 bpm, and temperature 36.2 °C. She received several injections of Paracetamol 1 g/8 h, Fentanyl 0.5 μg/kg/min, Ondansetron 4 mg/2 h, Tranexamate acid 1 g/8 h, and Ampicillin-Sulbactam 500 mg/8 h. After 2 days on a ventilator, she was extubated with better overall clinical outcomes.

## 3 Clinical discussion

Placenta previa is a condition that occurs in pregnancy when the placenta abnormally implants in the lower uterine segment, partially or completely covering the internal cervical orifice. It can be caused by several risk factors such as advanced maternal age, parity, maternal smoking, infertility treatment, previous C-section delivery, previous placenta previa, and repeated abortion [12]. Placenta previa occurs with an incidence of 0.3–0.5 % of all pregnancies. Placenta previa is also found in 20 % of APH which can cause death in mothers and newborns. It can lead to acute labor increasing maternal morbidity, uteroplacental insufficiency, premature birth, and perinatal death. C-section and blood transfusions are the only safe and appropriate ways of delivery [13].

A study showed that autologous transfusion provided better postoperative C-section and increased RBC, Platelets, HCT, and Hb compared to homologous transfusion [5]. Autologous transfusion effectively provides routine postoperative hemodynamics, electrolytes, and blood stability. However, it cannot completely replace allogenic transfusion [3].

In our patient, the blood loss is >1500 cc and our patient received four autologous blood bags with 450 cc each. No transfusion reaction and coagulopathy events were recorded. These conditions comply the requirements for the use of autologous transfusion. In addition to ensuring the sterility and contamination of the blood to be used and discontinuing the procedures if coagulopathy occurs autologous blood should be reinfused according to the volume lost and not given excessively [8]. Autologous transfusion in patients with placenta previa is sometimes not possible due to severe anemia, renal failure, bacteremia, and hypocoagulable [14].

**Fig. 2:**
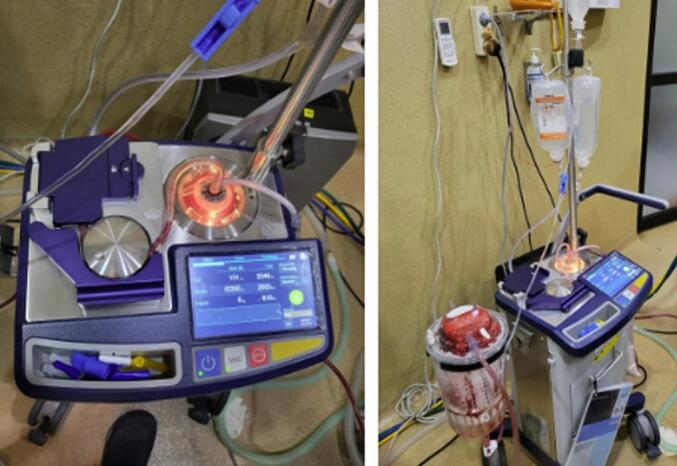
Autotransfusion or Cell Saver machine in our center that provides autologous blood during surgical procedures. In this case, the machine suctioned 2500 cc of blood. It used 1000 cc of normal saline as diluent and obtained 800 cc of packed cells. The machine also added 10,000 units of Heparin.

The use of an autologous transfusion device (Fig. 2) requires a minimum of 400–600 cc of blood. This blood can then be filtered, washed, concentrated, and transfused back to the patient. The blood is centrifuged and produces PRC. In addition, this process usually removes platelets and coagulation factors. However, it potentially results in dilutional coagulopathy particularly when it is used in standard intraoperative programs and with large blood volumes [15]. It occurs when blood is replaced by fluids that do not contain enough coagulation factors. It is typically described as the loss, consumption, or dilution of coagulation factors [16].

Despite the unwanted side effects, autologous transfusion has disadvantages in terms of the cost for storage and transportation. This storage requires extra monitoring, blood must be stored at a temperature of 1–6 °C, while when it is transported the temperature must be 1–10 °C. Postoperative plays an important role in achieving good outcomes [17]. Administration of multimodal analgesic drugs can reduce postoperative pain, this can be achieved through a combination of neuraxial opioid analgesia, oral analgesia, and peripheral nerve blockade [18].

In the use of autologous, RBC, Platelets, Hct, and Hb increased, but there was a decrease in the patient's IgA, IgG, and IgM [6]. Moreover, autologous transfusion decreases immunosuppression, and lowers the risk of infection, and transfusion response. As a result, it lessens the demand for allogenic blood supplies and permits safer transfusion for individuals with multiple auto-antibodies and rare blood types [5]. These are in line with our study in this case, where the patient's hemodynamic state improved after autologous transfusion in a patient with APH.

Health services require a physical-logistical support approach regarding this case. Generally, in Indonesian tertiary referral hospitals remain had inadequate infrastructure for autologous transfusion equipment and sub-specialist anesthesiology means that the use of autologous transfusion will be more difficult [8]. Furthermore, there is the potential for bacterial contamination of blood products compared to blood products which are usually more risky in autologous transfusion, requiring more advanced and costly quality of control standards to avoid bacterial contamination [19,20].

## 4 Conclusion

Autologous transfusion can be used in C-section patients with placenta previa. Autologous transfusion shows a prognosis free of side effects and transfusion reactions. Note that there must be careful and strict supervision of the autologous transfusion procedure, hemodynamic profile, and post-c-section management, especially anti-pain, antibiotic and anti-bleeding injections. Furthermore, this transfusion procedure must be under the supervision of an anesthesiologist and monitor adequate medical equipment. The weakness of autologous transfusion is that it is rarely practiced due to infrastructure requirements and cost-effective reasons. So, it is necessary to carry out further research in the form of experimental analytical clinical studies on populations and compare them with other types of transfusion approaches, for example allogenic.

## Source of support

The authors are grateful to all anesthesiologist of Dr. Moewardi General Hospital for providing feedback and technical support.

## Patient consent

Written informed consent was obtained from the patient for publication of this case report and accompanying images. A copy of the written consent is available for review by the Editor-in-Chief of this journal on request.

## Ethical approval

This case study was approved by Health Research Ethics Committee of Dr. Moewardi Hospital, No. 266/III/HREC/2023. Written informed consent was obtained from the patient for publication of this case report and accompanying images. A copy of the written consent is available for review by the Editor-in-Chief of this journal on request.

## Funding

This case study did not receive any specific grant from funding agencies in the public, commercial, or not-for-profit sectors.

## Author contribution

Rizki Dunniroh Kaukaba, Septian Adi Permana, Arif Zuhal Amin Hananto: study concept, imaging for this patient, surgical therapy for this patient. Faizal Muhammad: data collection, writing original draft preparation. Rizki Dunniroh Kaukaba: senior author and the manuscript reviewer. Septian Adi Permana, Arif Zuhal Amin Hananto: case preparation. All authors read and approved the final manuscript.

## Guarantor

Rizki Dunniroh Kaukaba, Faizal Muhammad.

## Research registration number

N/A.

## Conflict of interest statement

The authors declare that they have no competing interests.
